# Pseudonymization of patient identifiers for translational research

**DOI:** 10.1186/1472-6947-13-75

**Published:** 2013-07-24

**Authors:** Harald Aamot, Christian Dominik Kohl, Daniela Richter, Petra Knaup-Gregori

**Affiliations:** 1 NCT Trial Center, German Cancer Research Center, Heidelberg, Germany; 2 Institute of Medical Biometry and Informatics, Heidelberg University, Heidelberg, Germany; 3 Coordination Center for Clinical Trials (KKS), Heidelberg University, Heidelberg, Germany; 4 Translational Oncology, German Cancer Research Center, Heidelberg, Germany

**Keywords:** Pseudonymization, Pseudonymisation, Pseudonym, Anonyms and pseudonyms, Translational research, Biobanking, Data privacy, Individual research results, Record linkage, Re-identification, De-pseudonymization, De-pseudonymisation

## Abstract

**Background:**

The usage of patient data for research poses risks concerning the patients’ privacy and informational self-determination. Next-generation-sequencing technologies and various other methods gain data from biospecimen, both for translational research and personalized medicine. If these biospecimen are anonymized, individual research results from genomic research, which should be offered to patients in a clinically relevant timeframe, cannot be associated back to the individual. This raises an ethical concern and challenges the legitimacy of anonymized patient samples. In this paper we present a new approach which supports both data privacy *and* the possibility to give feedback to patients about their individual research results.

**Methods:**

We examined previously published privacy concepts regarding a streamlined de-pseudonymization process and a patient-based pseudonym as applicable to research with genomic data and warehousing approaches. All concepts identified in the literature review were compared to each other and analyzed for their applicability to translational research projects. We evaluated how these concepts cope with challenges implicated by personalized medicine. Therefore, both person-centricity issues and a separation of pseudonymization and de-pseudonymization stood out as a central theme in our examination. This motivated us to enhance an existing pseudonymization method regarding a separation of duties.

**Results:**

The existing concepts rely on external trusted third parties, making de-pseudonymization a multistage process involving additional interpersonal communication, which might cause critical delays in patient care. Therefore we propose an enhanced method with an asymmetric encryption scheme separating the duties of pseudonymization and de-pseudonymization. The pseudonymization service provider is unable to conclude the patient identifier from the pseudonym, but assigns this ability to an authorized third party (ombudsman) instead. To solve person-centricity issues, a collision-resistant function is incorporated into the method. These two facts combined enable us to address essential challenges in translational research. A productive software prototype was implemented to prove the functionality of the suggested translational, data privacy-preserving method. Eventually, we performed a threat analysis to evaluate potential hazards connected with this pseudonymization method.

**Conclusions:**

The proposed method offers sustainable organizational simplification regarding an ethically indicated, but secure and controlled process of de-pseudonymizing patients. A pseudonym is patient-centered to allow correlating separate datasets from one patient. Therefore, this method bridges the gap between bench and bedside in translational research while preserving patient privacy. Assigned ombudsmen are able to de-pseudonymize a patient, if an individual research result is clinically relevant.

## Background

### Next generation sequencing methods

Next generation sequencing methods have provided a wealth of new possibilities for the characterization of tumors in individual patients and laid the basis for new treatment options. However, under certain circumstances, the nature of the obtained data poses new risks to data privacy [1,2]. Patients that provide samples within translational research projects at the National Center for Tumor Diseases (NCT) Heidelberg therefore sign a specific informed consent accounting this risk. The consent specifically addresses the issue of data privacy, which would not be as critical, if the biological specimen were anonymized for sequencing and further analysis. However, translational research and personalized medicine ultimately require a two-way information highway from bench to bedside and vice versa [3,4]. Conducting research with anonymized data excludes the possibility of retrospective linkage to clinically relevant information, which is a fundamental ambition in biomedical translational research [3]. Without the possibility to de-pseudonymize a patient, he or she would be deprived of a direct benefit from research results. This is an ethical challenge, as under certain circumstances, individual research results in genetic and genomic research might lead to new treatment possibilities and therefore should be offered to study participants in a clinically relevant timeframe [5]. In a scenario where clinical follow-up data is later added to a biological sample to study outcome effects, anonymization is not applicable either. Furthermore, personalized medicine has to be based on the careful analysis of multifaceted data. By their very design next-generation-sequencing technologies and other high-throughput methods imply the involvement of many different persons and even external organizations in the data collection and analysis process, with all the corresponding additional risks to patient privacy [2,6]. To retain a semantic reference between patient and sample, while still complying with data privacy requirements, pseudonymization is the method of choice [7,8]. What we need is a data protection and privacy concept with solid pseudonymization and a streamlined de-pseudonymization process to transfer the results back to the clinic [9].

### Identity management

One prerequisite for pseudonymization is identity management. Current hospital information systems and clinical registries use patient registration modules which assign a unique patient identifier (PID) to a patient to constitute a mature identity management. This is a manual and elaborate task. Faldum et. al. [10] introduced an algorithm for automatic PID generation with optimal properties for error detection and correction which semi-automates identity management.

If hospital caregivers send biological samples and medical data to a sample processing lab (SPL) and a sequencing facility for genetic analysis, biospecimen like blood and tissue are annotated with contextual information like project name, tissue source site, sample, or study participant. Working with personal data is neither necessary nor acceptable at a SPL (although patients may have consented to processing of personal data where needed). Hence, a pseudonym (PSN) instead of personal data or the PID should be attached to a sample forwarded to a SPL. Consequently, a PID must be transformed into a PSN before a sample and its associated data leave the hospital for outside processing. This PSN must be repetitively unambiguous for a patient, so that researchers can correlate and utilize for secondary use several samples from the same patient (e.g. tumor and control sample). Additionally, there is a requirement for one or several persons who are able to compute back the PID from the PSN. These persons are called ombudsmen, because they safeguard the privacy of the patients while they are still able to identify them individually from a pseudonym, if necessary.

### Unambiguous pseudonyms

If record linkage at the research site is made with a pseudonym derived from a mature and stable PID, imprecision is low and error-tolerant record linkage mechanisms requiring personal information such as names and birthdates [11] are not needed. From a privacy point of view record linkage with a PSN is better than record linkage with personal information or a PID. For a controlled on demand aggregation of clinical data with translational data it must be possible to repetitively generate the identical PSN from a PID contained in the clinical data. Therefore, pseudonym generation must be repetitively unambiguous for all data sources [12]. If this requirement is fulfilled, a data warehouse for translational research objectives with records linked by a PSN can be considered [3,13,14].

### Duty separation of pseudonymization and de-pseudonymization

The person that pseudonymizes a patient’s sample is not necessarily the same person that performs the de-pseudonymization of the patient. Thus, a separation of duties regarding pseudonymization and de-pseudonymization will simplify the process. It is practical and reasonable to have one pseudonymization service provider and one or more flexibly chosen ombudsmen for de-pseudonymization tasks.

### Core requirements for translational pseudonymization

Generally speaking, the two core requirements for comprehensive pseudonymization in translational research are a repetitively unambiguous PSN for a given PID and a separation of duties regarding pseudonymization and de-pseudonymization of a patient. If warranted, ombudsmen should be able to de-pseudonymize a patient by a given PSN in a streamlined manner without further human interaction. An unambiguous PSN is essential to investigate correlations between phenotype and genotype of a patient.

In this paper we introduce a pseudonymization method which supports these core requirements of translational research. In addition, we present a software tool based on this method which is used in translational research projects at the NCT Heidelberg.

## Methods

We analyzed previously published pseudonymization approaches to evaluate whether or not they fulfill translational research requirements. In the process, we took characteristics of pseudonymization methods from the literature and used them to qualify the different approaches. These characteristics specifically address unambiguity of the pseudonyms and de-pseudonymization possibilities, which are the two core properties of pseudonymization for translational research. Given the characteristics, we arranged the different pseudonymization approaches into a table for a comparison of their features. Based on this feature matrix we constructed a method that possesses all desired characteristics displayed in the table and eventually implemented a prototype using the scripting language PHP and a Microsoft© SQL Server as database to prove the technical feasibility of this new method.

### Literature review

A PubMed database search revealed 10 hits for the keyword “pseudonymization” and 16 hits for the British English variant “pseudonymisation” (the search covered all database fields and was performed in August 2012). No duplicate records were found in all 26 documents, thus all documents found were screened for applicability. Six pseudonymization methods were identified and will be briefly presented below.

### Comparison of different pseudonymization approaches

To compare the different pseudonymization methods, we need to define the terms *pseudonymization* and *de-pseudonymization*. This is necessary, because the various approaches were initially developed for different scenarios and thus differ in their preconditions. For structural equality, we define the PID as a patient-identifying item to be able to compare two-step with single-step pseudonymization methods. Concerning de-pseudonymization, we define the following scenario: an ombudsman is a person independent from the pseudonymization process who wants to de-pseudonymize a patient (i.e. find out the PID from a given PSN). With this definition we can compare how many additional parties the ombudsman has to contact for a successful de-pseudonymization.

### Methodology by Neubauer et al

Neubauer et al. present a methodology for the pseudonymization of medical data [15-17]. Their methodology makes it possible to reestablish the relationship between a patient and her/his samples by an authorized user. An authorized user can create sets of PSNs, so-called shared PSNs, to connect patients to samples for research purposes. While providing perfect patient privacy, this method implies assigning a new PSN to every sample of a patient. This fact prevents the reuse of the PSN to integrate bedside and biology and to bridge the gap between clinical care and medical research in a patient-centric manner [3]. The methodology of Neubauer et al. is a very innovative concept to control authentication, authorization and access to medical data and provides perfect privacy, but lacks an unambiguous pseudonymization transformation of a patient identifier for secondary use. It would need to be added to their methodology to fulfill translational research requirements.

### Method by Noumeir et al

Noumeir et al. define two types of pseudonymization [18]: pseudonymization with one-way pseudonyms (which cannot be reversed) and reversible pseudonymization. One-way pseudonyms are applicable for translational research with a specific scientific question. They are not appropriate, however, if personalized treatment decisions based on systematic or incidental findings [5] are considered. Therefore one-way pseudonyms are not analyzed in this manuscript. Reversible pseudonymization, though, offers the possibility of re-identification by either mapping tables or mapping functions. As this mapping has to be secured, Noumeir et al. propose a symmetric encryption scheme. As a matter of fact, re-identification has to be restricted by either hardware/technical or organizational measures as the IT system implementing the method can both encrypt and decrypt the mapping information. This permits outsourcing of the pseudonymization service although it has to be combined with exhaustive legal provisions prescribing which ombudsman is authorized to de-pseudonymize which patients. The service provider then has to refer to the contract for every single de-pseudonymization request, providing documented evidence that the requester is entitled to de-pseudonymize the patient from the given pseudonym. This turns de-pseudonymization into a complex and bureaucratic process with human interaction.

### Method by Pommerening et al

**Figure 1:**
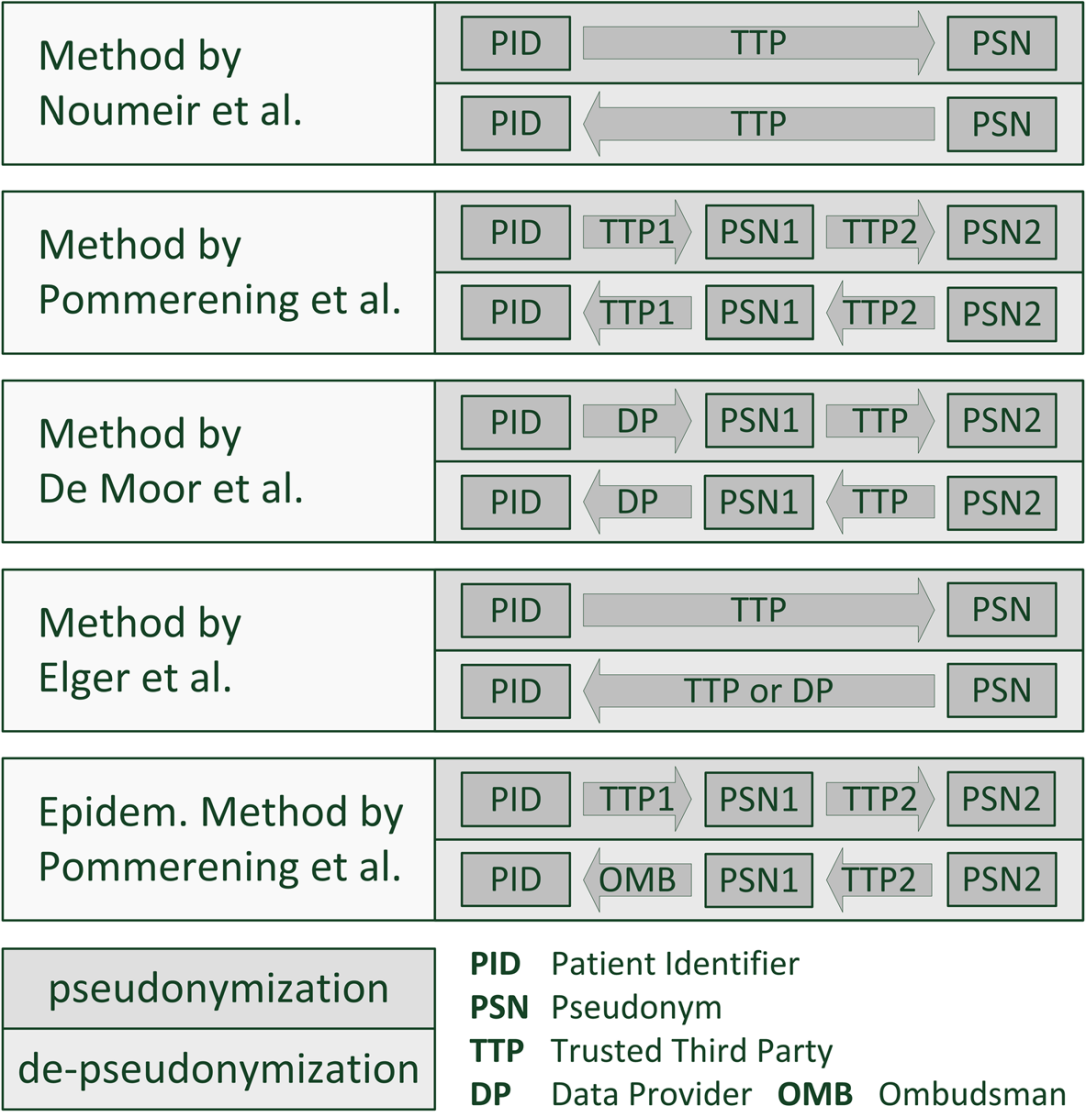
**Pseudonymization methods.** The five applicable pseudonymization methods which were identified in a PubMed database search. Their functional principles are simplified for faster perception.

Pommerening et al. present a method for the pseudonymization of patient samples which is generally accepted among German data privacy advocates [19,20]. The method is called “the pseudonymous research database” or “model B of the generic data privacy concept of the TMF e. V.” [20]. The method involves two service providers called trusted third parties (TTPs), who act as service providers and are employed for both, pseudonymization and de-pseudonymization of the patient. Pommerening et al. do not consider the PID a patient-identifying item like we do. They define patient identifying data (IDAT), from which a PID is derived by TTP1 (first pseudonymization). From the PID, a PSN is derived by TTP2 (second pseudonymization). We have translated their terminology (IDAT → PID → PSN) into our terminology (PID → PSN1 → PSN2) to facilitate comparison of their method with the other methods (see Figure 1). Their method allows reuse of the PSN for further research as described by Prokosch et al. [3], because only one pseudonym (PSN2) is assigned per patient. The method can also be applied to research data exported from a single source information system for patient care and research [21]. Nevertheless the de-pseudonymization of a patient requested by an authorized ombudsman involves two additional parties (TTP1, TTP2) with human interaction and thus appears difficult to organize as a swift routine de-pseudonymization process.

### Method by De Moor et al

De Moor et al. emphasize the role of a TTP for pseudonymization [22-25]. They identify the threat of an unauthorized re-identification by dictionary attacks and therefore introduce a pre-pseudonymization step preceding the pseudonymization by a TTP. This approach differs from the method of Pommerening et al. in having the data provider rather than another TTP perform the pre-pseudonymization. This also simplifies the entire process, because only one TTP instead of two is needed for pseudonymization. Still, de-pseudonymization of a patient by an authorized ombudsman requires two additional parties: the TTP and the data provider that performed the pre-pseudonymization step.

### Method by Elger et al

Elger et al. [26,27] define two reversible pseudonymization techniques: reversible single-pass and reversible dual-pass. The reversible dual-pass technique uses two pseudonymization steps to safeguard patient privacy and therefore corresponds to the methods proposed by Pommerening et al. and De Moor et al. The reversible single-pass method allows de-pseudonymization in a single step and, while being more straightforward, requires additional security measures, such as symmetric or asymmetric encryption, when compared to the double-pass technique. The authors identify asymmetric encryption as a technique that allows for a separation of duties concerning pseudonymization and de-pseudonymization. Nevertheless, their proposed pseudonymization method implements a symmetric encryption with a key for each clinic taking part in their research project. Their method is similar to the method by Noumeir et al. but due to clinic-specific keys their de-pseudonymization process can be organized with less bureaucratic hassle. This is achieved by enabling each clinic, apart from the pseudonymization service provider, to de-pseudonymize their patients through their clinic-specific key.

### Epidemiological method by Pommerening et al

The pseudonymization procedure for German epidemiological cancer registries by Pommerening et al. defines a concept for data flow and storage for population-based cancer registries [28]. A trusted office (TTP1) receives patient identifying data (IDAT) plus payload data and generates a patient identifier (PID). This PID and the payload data are then sent to a registration office (TTP2) which generates a pseudonym (PSN) from the PID and stores it with the data. We translate the terminology of this method (IDAT → PID → PSN) into our terminology (PID → PSN1 → PSN2). The trusted office (TTP1) simultaneously encrypts the IDAT with an asymmetric key and sends the cipher along with PSN1 to a supervising office (OMB). This supervising office has the private key for decryption, hence is the ombudsman in our terminology. This way, the powers of de-pseudonymization are separated between the registration office (TTP2) and the supervising office (OMB). The supervising office (OMB) cannot directly de-pseudonymize a person with a given PSN2. It has to request computation of the PSN1 from the registration office (TTP2) first, as this is the only way to reveal the PID.

### Methods characteristics comparison

Except for the methodology by Neubauer et al. all methods presented above are suitable for personalized oncology research programs as they perform pseudonymization and fulfill data privacy requirements. Elger et al. define two characteristics to distinguish between different pseudonymization techniques:

First: Pseudonymization can be computed back (=reversible) or not (=one-way).

Second: Pseudonymization is performed once (=single-pass) or twice (=dual-pass).

Taking de-pseudonymization into account, further characteristics have to be added:

Can the duties of de-pseudonymization and pseudonymization be separated?

How many additional parties must be contacted for de-pseudonymization?

Separation is not possible with symmetric encryption schemes (duty-united), while asymmetric encryption schemes do allow a separation of duties [26]. A party different from the pseudonymization service provider can be appointed to de-pseudonymize a patient from a pseudonym (duty-separated). None of the presented methods perform a reversible pseudonymization in combination with a direct duty-separated de-pseudonymization by a party other than the pseudonymization service provider (see Table 1). The authors therefore propose a new pseudonymization method with an asymmetric encryption scheme: the reversible single- or dual-pass pseudonymization method combined with duty-separated de-pseudonymization, or briefly – translational pseudonymization method.

**Table 1:** Characteristics of the different pseudonymization methods

**Method**	**Reversible?**	**Repetitively un-ambiguous and patient-centric?**	**Single-pass pseudony-mization?**	**Dual-pass pseudonymization?**	**Duty-separated regarding de-pseudonymization?**	**Additional parties needed for a de-pseudonymization?**
**Neubauer et al.**	X	-	X	X	X	n.a.
**Noumeir et al.**	X	X	X	-	-	1
**Pommerening et al.**	X	X	-	X	-	2
**De Moor et al.**	X	X	-	X	-	2
**Elger et al.**	X	X	X	-	-	1
**epidemiological**	X	X	-	X	(X)*	1
**translational**	X	X	X	(X)**	X	0

* power-separated, but not duty-separated.

** not realized in our project.

## Results

### Translational pseudonymization method

**Figure 2:**
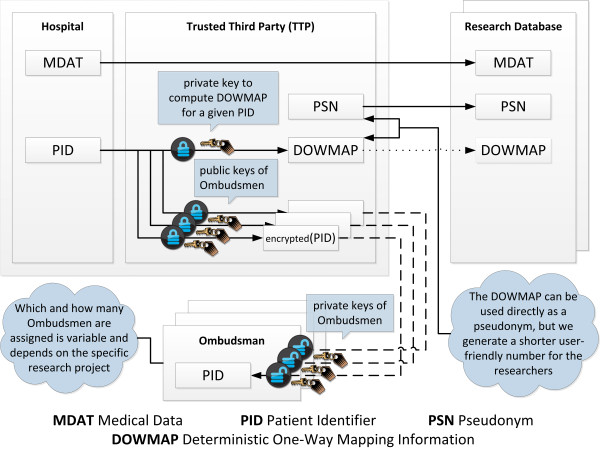
**Logical concept of translational pseudonymization.** The idea is to use an asymmetric encryption scheme within the pseudonymization. Therefore, nobody but the keeper of a corresponding secret private key can de-pseudonymize a patient.

In order to streamline pseudonymization in translational research projects, we suggest a pseudonymization technique with duty-separated de-pseudonymization of the patient carried out directly by dedicated, named ombudsmen. This can be achieved using an asymmetric encryption scheme (see Figure 2).

**Figure 3:**
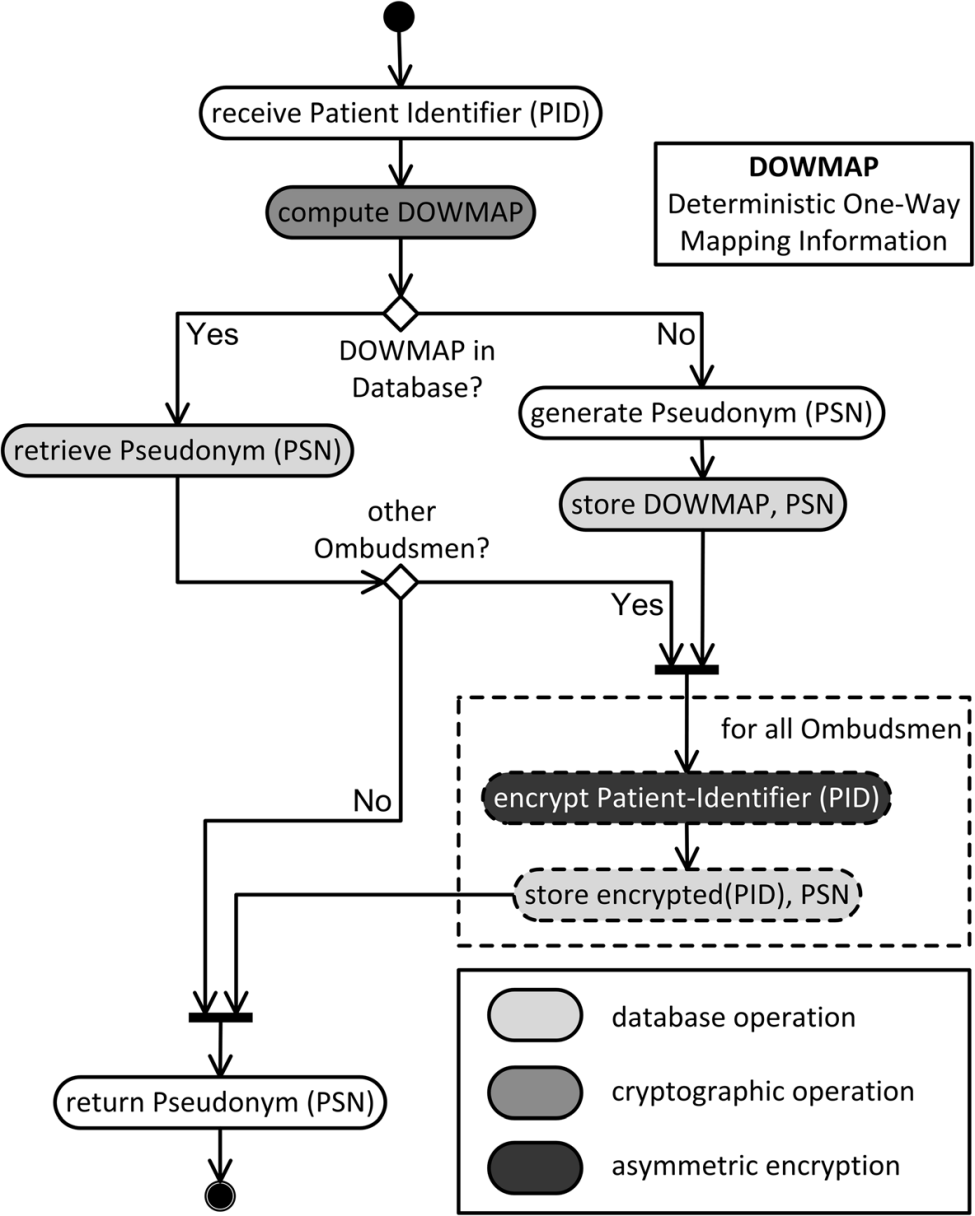
**The translational pseudonymization algorithm.** The algorithm has two scenarios. Either a new pseudonym is created or the patient has been pseudonymized before and his pseudonym is retrieved from the database.

We start with the pseudonymization procedure. The operations carried out by the TTP for a pseudonymization are shown in Figure 3. When the pseudonymization service receives a PID, it first computes deterministic one-way mapping information (DOWMAP) for a PID.

After that the operating mode of the pseudonymization service branches into two scenarios. In case of an initial PID pseudonymization, a handy pseudonym (PSN) is generated. In case of a repeated pseudonymization, the PSN can be retrieved from the database. Once the PSN is available, it is possible to encrypt the PID multiple times with the public keys of different ombudsmen allowing each of them to de-pseudonymize the patient with his or her own private key.

**Figure 4:**
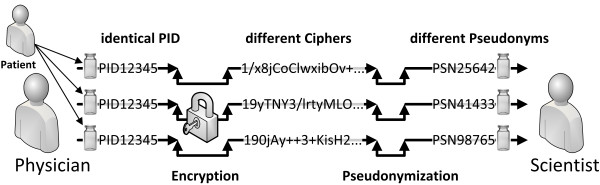
**The determination problem with approved encryption algorithms.** Approved encryption algorithms utilize a cryptographically secure, pseudorandom initialization vector which renders ambiguous the resulting cipher of the encryption operation of a distinct input. Therefore, it cannot be used directly to create an unambiguous pseudonym.

The computation of DOWMAP needs close attention. One could suggest using an encrypted PID directly as a pseudonym, but this approach causes a determination problem: Encryption algorithms generally produce different ciphers when used repeatedly on the same given input. This circumstance is illustrated in Figure 4. This problem applies to all encryption algorithms approved under the Technical Directive TR-03116 for eCard projects of the German Federal Government [29] and various other international directives. Technically speaking, all symmetric and asymmetric encryption operations performed in a block cipher mode with additional entropy (e.g. initialization vector) produce different ciphers for the same given input. Solely the Electronic Code Book (ECB) mode does not add additional entropy and therefore always produces the same cipher on a given input. This is the very reason why ECB mode is considered less secure and why it is not approved under TR-03116. For our translational research scenario we need one single deterministic and distinct pseudonym per patient which can be applied to clinical data for secondary use in a research database. As we wanted to apply only approved methods, we chose not to use an unambiguously encrypted PID in ECB mode as a directly-derived pseudonym.

**Figure 5:**
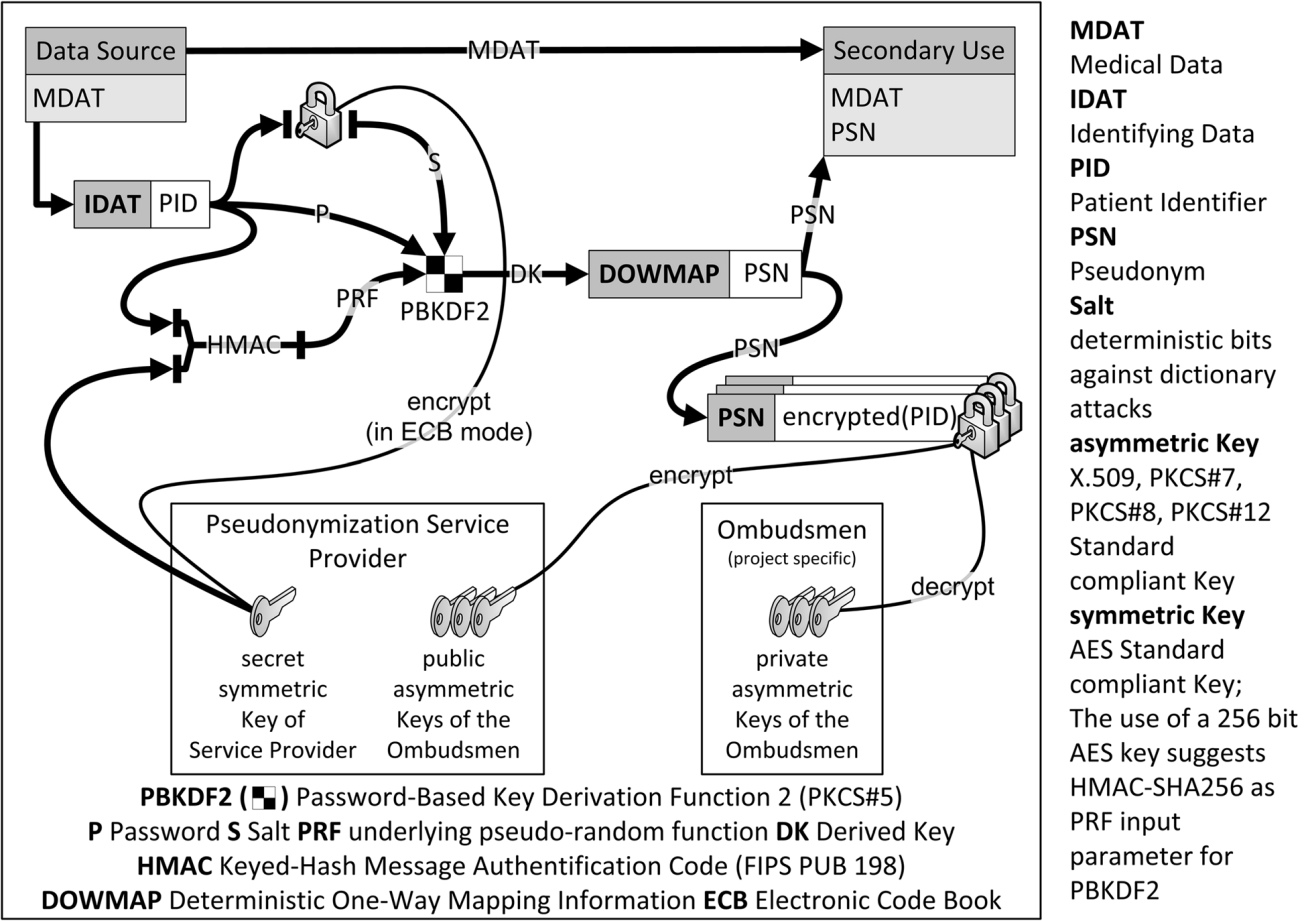
**The technical solution for the determination problem.** Deterministic One-Way Mapping Information (DOWMAP) is used to unambiguously determine, if a patient has been pseudonymized before. This is accomplished with cryptographic hashing and symmetric encryption. Asymmetric encryption is performed after this mapping step.

Instead, we chose an elaborate procedure to compute the deterministic one-way mapping information (DOWMAP). The most straightforward DOWMAP would be a Keyed-Hash Message Authentication Code (HMAC) [30]. HMAC incorporates a cryptographic key [31] to secure against dictionary attacks. A better DOWMAP is the “Password-Based Key Derivation Function 2” (PBKDF2) [32], as it adds entropy (called salt) and a computing effort (called iteration count) to a one-way function like the HMAC. PBKDF2 increases the computing cost for an insider dictionary attack in a linear way and can make attacks with rainbow tables [33,34] uneconomical. We apply the PBKDF2 with a deterministic salt (HMAC of the PID) to address the determination problem in obtaining a Derived Key (DK) which constitutes our DOWMAP (see Figure 5). Subsequently, we generate a shorter, more user-friendly pseudonym (PSN) for better practicability in the lab.

For de-pseudonymization, an ombudsman should use a client software application. This allows keeping the private key of the ombudsman - the primary aim for attackers – local. With a client application, the ombudsman exclusively communicates his public key (certificate) and the relevant PSN to the pseudonymization service provider (TTP). If the TTP does have a corresponding encrypted PID, it is automatically transferred to the ombudsman. With her/his private key the ombudsman may then decrypt the encrypted PID using his local application. The ombudsman thus only keeps and safeguards her/his private key and does not have to manage a list of PID-PSN relations, which are stored by the pseudonymization service provider (TTP). Since the PID is asymmetrically encrypted, these relations are useless for everybody but the ombudsman.

### Pseudonymization service for the NCT trial center

A functional software service was programmed to prove the technical feasibility of this new translational pseudonymization method. It is in production use for 28 translational research projects at the Heidelberg Center for Personalized Oncology. These projects stretch across various cancer types (acute myeloid leukemia, multiple myeloma, lung cancer, glioblastoma, gastric cancer, mammary carcinoma, soft tissue sarcoma, head- and neck squamous cell carcinoma, melanoma, pancreatic cancer and some more) and have different patient cohort sizes ranging from a handful up to several hundreds. The service is implemented in PHP, a scripting language which can be embedded into HTML and is interpreted by a hypertext preprocessor. It supports object-oriented programming and can be combined with scripting and rapid web development. PHP is used in reliable clinical applications, such as drug information systems [35]. A Microsoft© SQL Server was chosen as database, but thanks to a database abstraction layer most relational database management systems are supported.

**Figure 6:**
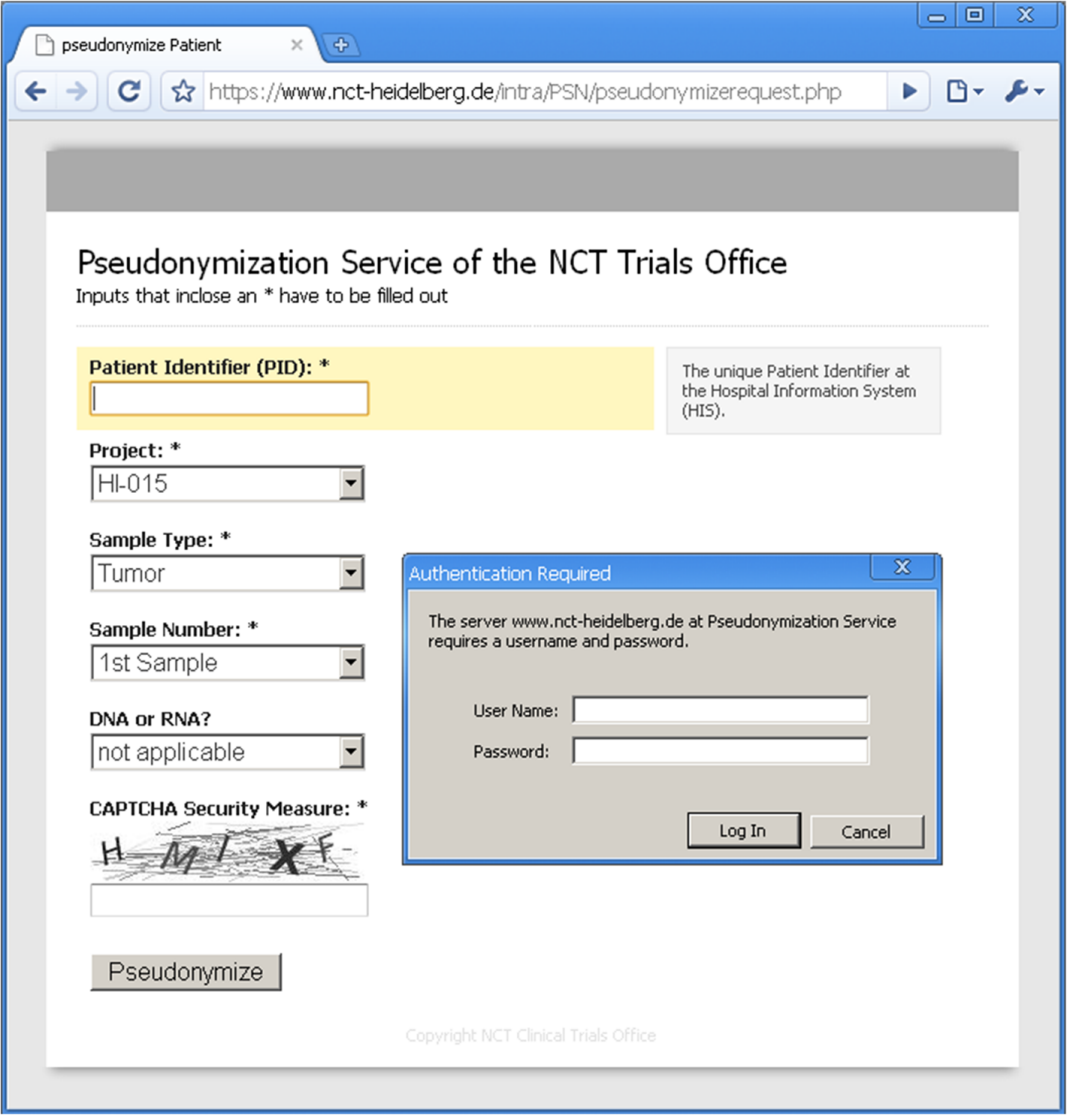
**The software prototype – single sample pseudonymization request.** The PID is the only input to create or retrieve a pseudonym. Selection of a research project controls which ombudsmen are able to de-pseudonymize a patient. Other inputs are used to generate a comprehensive barcode for sample storage management within a “Laboratory Information Management System” (LIMS). The CAPTCHA and the authentication screen are also visible on the screenshot.

**Figure 7:**
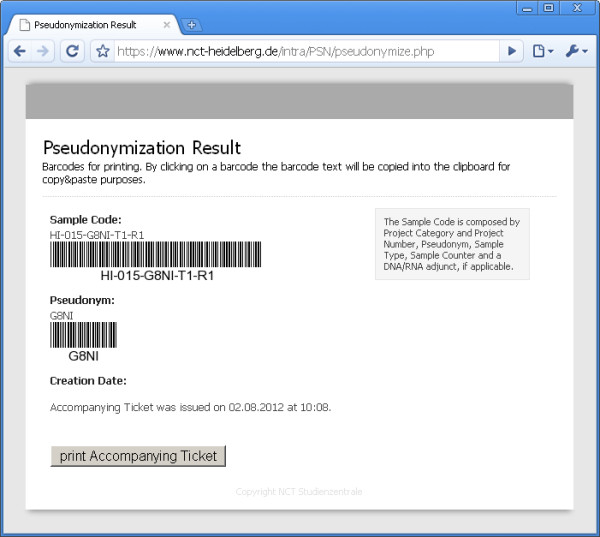
**The software prototype – single sample pseudonymization result.** The pseudonymization service creates an accompanying ticket that can be printed out and attached to a patient sample. The ticket contains the patient’s pseudonym and a sample barcode used at the sample processing lab as an identifier for the LIMS. The LIMS is used to document storage, processing and quality control of the samples according to common good laboratory practice (GLP).

The service is used for translational research projects in Heidelberg. Loss of an ombudsman’s key or PIN renders de-pseudonymization virtually impossible. As our method enables us to have more than one cryptographic public key, we appointed an institutional review board as a “backup ombudsman” in addition to various project-specific ombudsmen. Typically, the principal investigator (PI) of a project serves as an ombudsman. After institutional authentication, authorized users access the pseudonymization service through a web-based interface. Either single sample pseudonymization (see Figure 6) or batch pseudonymization with a spreadsheet upload function are available. Batch sizes are limited to minimize the risk of security issues with malicious service abuse. The service’s authentication mechanism relies on the “Lightweight Directory Access Protocol” (LDAP) querying the institutional domain server. Against service abuse, a “Reverse Turing Test” RTT is performed by the “Completely Automated Public Turing test to tell Computers and Humans Apart” (CAPTCHA), developed by Ahn, Blum and Langford [36]. In our specific prototype it displays random characters in a graphic that can’t be interpreted by a machine. The simplest way of pseudonymization in our prototype is to generate an accompanying ticket with a barcode containing the patient’s pseudonym (see Figure 7). This ticket can be printed out and sent to the sample processing lab with a biospecimen. Batch-pseudonymization with spreadsheets is available for pseudonymization of several biospecimen gained at once, e.g. from the NCT tissue bank for a specific research project.

## Discussion

Our method to pseudonymize patient identifiers offers a separation of duties for pseudonymization and de-pseudonymization and generates a repetitively unambiguous pseudonym for a patient. We combined these two features to address specific requirements in translational research projects. It is difficult to balance data privacy protection rights with a patient’s right to learn about individual research results. In the end, any translational research sample needs a unique, patient-centric identifier. The person gaining the sample, the research project and the scientific objectives each determine the point when any identifier is applied to a patient’s sample. Our method offers a global solution for repetitively unambiguous pseudonymization of this identifier for the purpose of translational research. The duty of de-pseudonymization can then be handed over to an independent party. Patients participating in a translational research project will have to accept the direct de-pseudonymization option [5]. Actual de-pseudonymization will require more than just notifying the treating physician. All data of the case will have to be discussed at a molecular tumor board comprising clinicians, bioinformaticians and the principal investigators of the research projects affected. The board will then suggest options for the patient and support the clinician in interpreting the results for the patient. To prepare for ethically indicated de-pseudonymization or key loss, the following entities should be considered for the role of ombudsmen: institutional review boards, ethics committees or data security officers. Before her/his assignment, any ombudsman has to undergo scrutiny to ascertain that she/he fulfills legal requirements. Under German standards, for instance, a doctor would qualify as an ombudsman, if he is treating patients in a translational research program with a corresponding informed consent.

From a mathematics and information theory point of view, a pseudonymization transformation should be an injective or at least collision-resistant function. The need to protect mapping information makes any technique other than a cryptographic function largely unlikely. Therefore, the authors of this article deliberately chose to evaluate encryption, decryption and cryptographic one-way-functions only. This is not meant to imply that the usefulness of other functions or techniques is beyond imagination.

### Field report on pseudonymization and de-pseudonymization activities

The usability of the NCT pseudonymization service is acceptable, because users do not need a separate username and password, but may authenticate themselves with their institutional login and password. The pseudonymization of single biospecimen after surgery or blood withdrawal works fine and happens on an incidental basis. A pseudonymization receipt with a barcode is printed out and used to document the sample processing and storage. Working with a barcode scanner eliminates manual documentation errors. For batches, the Sample Processing Lab (SPL) uses a sample submission spreadsheet. Once it is filled out by the principal investigator of the project, it can be uploaded to the pseudonymization service for batch pseudonymization. The resulting submission sheet with pseudonyms (text and barcode) is printed out for further processing and storage documentation according to good laboratory practice (GLP).

Ethically or medically indicated de-pseudonymization has not taken place so far, but successful de-pseudonymization was performed by a principal investigator. This was done due to suspected manual confusion of two samples during entry into the sample submission sheet. In order to track the entire submission process, the investigator, among other measures, de-pseudonymized the pseudonyms for the two samples. He was able to reconstruct the pseudonymization process and to verify the right relation between patients and samples. The NCT Trials Office as a service provider would not have noticed the activity, if they had not checked the audit logs of the pseudonymization service (“User X has performed the de-pseudonymization of patient with pseudonym Y”). This shows that both pseudonymization and de-pseudonymization processes will function without technical support from the service provider. These are our experiences so far and some ombudsmen are excited that they now have obtained an asymmetric key pair which is used analogous to secure e-mail exchange (S/MIME).

If a researcher makes a finding that could possibly indicate a de-pseudonymization, the subsequent steps are defined by a Workgroup called “Ethical and Legal Aspects of Whole Genome Sequencing” (EURAT). EURAT is a project on normative issues of total genome sequencing that brings together scholars from Heidelberg University, the Heidelberg University Hospital, the German Cancer Research Center (DKFZ), the European Molecular Biology Laboratory (EMBL), the Max Planck Institute for Comparative Public Law and International Law and the Research Center for Health Economics at the Hannover University [37]. Primary results of the workgroup are a codex for non-clinical researchers and two templates for patient information and informed consents. Also a process for de-pseudonymization in case of normal or incidental findings is defined. If a researcher has a finding, he can hand it over to an interdisciplinary board. Depending on the finding the researcher can choose between the “board for normal findings” and the “board for incidental, additional, or difficult findings”. The board discusses the case and decides whether the de-pseudonymization will be performed or not. If yes, the ombudsman performs the de-pseudonymization and then matches the PID with the name of the patient and his treating physician. The treating physician or alternatively a board member gets informed by the ombudsman and then can deliver the results to the patient. In case the board needs additional clinical information from the patient to reach a decision, the ombudsman gathers this information in cooperation with the treating physician. That is especially why the ombudsman plays an important role in our translational pseudonymization method. Of course, all persons involved into the process are required to observe strict confidentiality and data protection.

### Limitations

Encryption of the medical payload data is not implemented in our software prototype. The asymmetric encryption scheme we use implies a high computational cost. Therefore, symmetric schemes for encryption of the payload data appear more reasonable, especially for large quantities of data (big data). In addition, genetic data bears an inherent re-identification risk [1,2]. This could be addressed with a symmetric cryptographic approach as proposed by Cassa et al. [38]. In general, access to genetic data has to be secured by appropriate authentication and authorization methods to control the inherent re-identification risk.

Alternatively, our method could be incorporated or combined with a comprehensive health data management concept like the one presented by Neubauer et al. [17] to provide encryption of the medical payload data. However, this would require nationwide availability of an electronic health card infrastructure and health care professional cards. This is currently not the case in Germany and other countries, but planned for the future.

In our translational research projects, patient identities are managed within the Heidelberg university hospital information system. A multi-center translational research project will require an identity management system superior to that of a hospital information system. Multi-center identity management and pseudonymization could be made available by combining the methods and concepts that Faldum and Pommerening propose [10] with our translational pseudonymization method.

**Figure 8:**
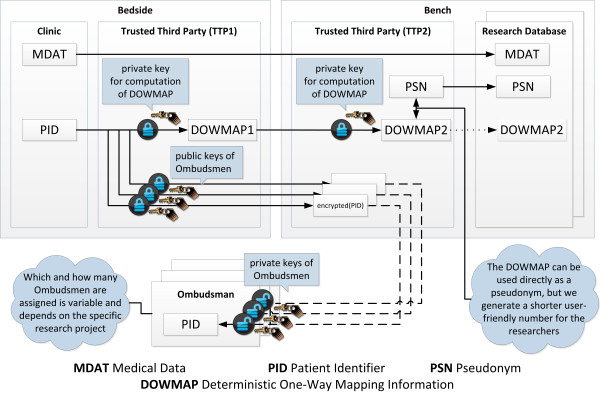
**Logical concept of translational dual-pass pseudonymization.** The informational separation of powers achieved with two trusted third parties is combined with a separation of duties regarding a direct de-pseudonymization by authorized ombudsmen.

We did not yet implement a dual-pass pseudonymization in our project, but we plan to upgrade our technique to pseudonymize with a dual-pass method similar to the ones Pommerening et al. and De Moor et al. propose (see Figure 8). That would address large projects with extraordinary safeguards. Such projects are for example large distributed long-term projects with biobanking, several institutions involved, clinical data warehouses, and personalized medicine genome sequencing projects. The benefits would be a reversible, repetitively unambiguous, dual-pass pseudonymization which still allows appointed ombudsmen to directly de-pseudonymize a patient. Combined with a sustained consent management and defined de-pseudonymization processes this is a promising approach. In a two-step setting, the first pseudonymization step will be performed by the clinical sample gainer who then tags the biological sample with the first PSN. The researcher receiving the sample then performs the second pseudonymization step to obtain the second PSN for research. The burden of pseudonymizing a sample twice results in informational separation of powers and therefore enhanced data privacy. The first PSN serves as feedback code for the sample gainer who knows the patients’ identity and labels the sample. The second PSN is applied by the researcher receiving the sample and can be disclosed to colleagues working in the research context with the sample.

Pseudonymization alone does not eliminate the need to define an appropriate access policy to the bulk of research data [39]. Data propagation and aggregation with external data sources always bears a re-identification risk. The principal investigator of a research project has to weigh the protection of personal health information against the option to join the data with other data sources through a pseudonym. Fullerton and Lee propose a data access committee or similar internal oversight body with suitable stakeholder representation to support the principal investigator in assessing the risk-to-benefit ratio of data linkage [40]. Institutional review boards or ethics committees are another instrument to assist in the decision, whether such data conflation is ethical and justifiable.

### Threat analysis for the method and its software prototype

A threat analysis was performed to evaluate the possibilities and risks for unauthorized re-identification of a patient. The following threats to the newly introduced pseudonymization method have been identified:

•Database theft

•System or hardware theft

•Social engineering

•Malicious service use

•Malicious insiders

Database theft has no negative impact, because any deterministic mapping information is secured by the secret key of the pseudonymization service provider. The only information visible to the attacker in plaintext is which and how many pseudonyms have been created. Even a cryptanalysis will not yield any output, because the cryptographic keys are not available to the attacker.

A system or hardware theft is a serious threat. The secret key of the pseudonymization service provider and all data from the database would be vulnerable to a dictionary attack. A dictionary attack with lookup or rainbow tables [33] can be rendered uneconomical by applying a deterministic salt to the HMAC. The computing cost of a dictionary attack by an insider with the secret key can only be controlled in a linear way, e.g. by applying the PBKDF2 function. If the iteration parameter of PBKDF2 is chosen high enough, computing cost will remain too expensive. On the other hand, if the iteration parameter is chosen too high, the pseudonymization process is too time-consuming and bulky. Ultimately, it is critical that the hardware is secured against physical theft and that IT systems are protected with standard and up-to-date IT security measures against hijacking or other control-gaining attacks. It seems highly advisable to use a hardware security module to store the secret key of the pseudonymization service. A high iteration parameter for PBKDF2 and security measures against physical theft and hijacking will provide effective security against this threat.

Social engineering targeting the ombudsmen is a serious threat. Preventive measures must primarily include, but should not be limited to, educating the ombudsmen so they are aware of their sensitive and critical role. An additional technical measure is for them to apply a personal identification number (PIN) or passphrase to protect their secret private asymmetric keys. Both measures combined provide reasonable protection against this threat.

Malicious service use is a possible threat to the method. Repetitive, high frequency access to the pseudonymization service by a specific machine in order to guess PID-PSN relations must be denied. An authentication procedure to gain access to the service will at least prohibit unauthorized access. Repetitive service usage or abuse can be prevented by a "Reverse Turing Test" (RTT) [41]. The test helps determine if a machine or a human is trying to use the service and will eventually block the attacking machine from service use. A human is not fast enough to perform a service abuse attack. Therefore, a RTT and authentication and authorization mechanisms are appropriate security measures against this threat.

Malicious insiders are a threat that may not be underestimated. Especially a sabotage of the pseudonymization system is a threat that must be addressed by credible and loyal staff. Solely a two-step pseudonymization method yields protection against sabotage of untrustworthy staff.

Further technical threats may apply for any actual implementation of the pseudonymization method. In the pseudonymization service of the NCT Trial Center we have addressed:

•Traffic interception

•Database attacks

Transport Layer Security“ (TLS) establishes an encrypted connection between the service user and the service to prevent threats by traffic interception. Man-in-the-middle attacks are not addressed, because the service is offered exclusively in a trusted institutional intranet with appropriate authentication and authorization mechanisms. Therefore, it is inaccessible to external attackers trying to impersonate the service users.

Database attacks with SQL injections are impossible. Any data received by the service is encrypted and escaped by a database abstraction layer before it is stored in the database.

The authors identified no other malicious code or attempts to attack this software service.

## Conclusions

With the method presented above investigators can efficiently solve data privacy problems and streamline patient-centric research conducted in cooperation with external institutions. The biunique link between patient and pseudonym facilitates data aggregation over various data sources for translational research objectives or data warehousing approaches. All things considered, our method provides a secure and applicable approach for the complete circle of patient data pseudonymization and de-pseudonymization in translational research projects. It will be a valuable tool for personalized medicine projects – on the Heidelberg campus and beyond.

## Abbreviations

ECB: Electronic code book; HMAC: Keyed-hash message authentication code; LDAP: Lightweight directory access protocol; LIMS: Laboratory information management system; NCT Heidelberg: National Center for Tumor Diseases Heidelberg; PBKDF2: Password-based key derivation function 2; PID: Patient identifier; PIN: Personal identification number; PSN: Pseudonym; RTT: Reverse Turing test; SPL: Sample processing lab; TLS: Transport layer security; TTP: Trusted third party.

## Competing interests

The technology transfer office of the German Cancer Research Center has evaluated the method and issued a patent application. The authors declare that they have no other competing interests.

## Authors’ contributions

HA invented the asymmetric pseudonymization method and implemented the software prototype. CK helped to discuss and refined the graphics. PK and CK helped to draft and revised the manuscript. DR wrote background and conclusions regarding translational research projects and objectives. All authors have read and approved the final manuscript.

## Pre-publication history

The pre-publication history for this paper can be accessed here:

http://www.biomedcentral.com/1472-6947/13/75/prepub
